# 4-Hy­droxy-2-[(4-iodo­benzo­yl)meth­yl]-3-(3-meth­oxy­benzo­yl)-2*H*-1,2-benzothia­zine 1,1-dioxide

**DOI:** 10.1107/S1600536810032265

**Published:** 2010-08-18

**Authors:** Salman Gul, Hamid Latif Siddiqui, Matloob Ahmad, Masood Parvez

**Affiliations:** a Institute of Chemistry, University of the Punjab, Lahore 54590, Pakistan; b Applied Chemistry Research Centre, PCSIR Laboratories Complex, Lahore 54600, Pakistan; c Department of Chemistry, University of Calgary, 2500 University Drive NW, Calgary, Alberta, Canada T2N 1N4

## Abstract

In the title mol­ecule, C_24_H_18_INO_6_S, the heterocyclic thia­zine ring adopts a half-chair conformation, with the S and N atoms displaced by 0.381 (5) and −0.449 (5) Å, respectively, from the plane formed by the remaining atoms in the ring; the puckering parameters are *Q* = 0.550 (2) Å, θ = 61.7 (2)° and ϕ = 31.4 (3)°. The conformation is stabilized by an intra­molecular O—H⋯O hydrogen bond. The two nonfused benzene rings lie almost parallel to each other [dihedral angle = 9.18 (4)°], with a separation of 3.754 (2) Å between the centres of gravity of the two rings, indicating strong π–π inter­actions.

## Related literature

For biological applications of benzothia­zines, see: Lombardino & Wiseman (1972 ▶); Zinnes *et al.* (1982 ▶); Zia-ur-Rehman *et al.* (2005 ▶); Turck *et al.* (1996 ▶); Ahmad *et al.* (2010 ▶). For crystal structures of related compounds, see: Siddiqui *et al.* (2008 ▶); Gul *et al.* (2010 ▶). For puckering parameters, see: Cremer & Pople (1975 ▶).
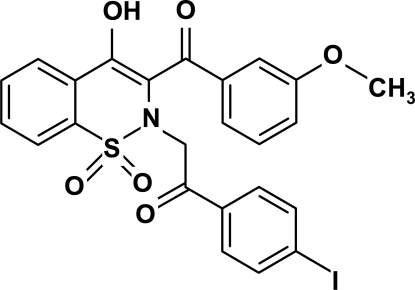


## Experimental

### 

#### Crystal data

C_24_H_18_INO_6_S
*M* *_r_* = 575.35
Monoclinic, 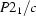

*a* = 9.7392 (2) Å
*b* = 11.5288 (3) Å
*c* = 20.4634 (4) Å
β = 100.5288 (11)°
*V* = 2258.97 (9) Å^3^
*Z* = 4
Mo *K*α radiation
μ = 1.55 mm^−1^
*T* = 173 K
0.12 × 0.10 × 0.08 mm

#### Data collection

Nonius KappaCCD diffractometer
Absorption correction: multi-scan (*SORTAV*; Blessing, 1997 ▶) *T*_min_ = 0.836, *T*_max_ = 0.886
16229 measured reflections
3960 independent reflections
3620 reflections with *I* > 2σ(*I*)
*R*_int_ = 0.017

#### Refinement

*R*[*F* ^2^ > 2σ(*F* ^2^)] = 0.026
*wR*(*F* ^2^) = 0.061
*S* = 1.08
3960 reflections
300 parameters
H-atom parameters constrained
Δρ_max_ = 0.76 e Å^−3^
Δρ_min_ = −0.83 e Å^−3^

### 

Data collection: *COLLECT* (Nonius, 1998 ▶); cell refinement: *DENZO* (Otwinowski & Minor, 1997 ▶); data reduction: *SCALEPACK* (Otwinowski & Minor, 1997 ▶); program(s) used to solve structure: *SHELXS97* (Sheldrick, 2008 ▶); program(s) used to refine structure: *SHELXL97* (Sheldrick, 2008 ▶); molecular graphics: *ORTEP-3 for Windows* (Farrugia, 1997 ▶); software used to prepare material for publication: *SHELXL97*.

## Supplementary Material

Crystal structure: contains datablocks global, I. DOI: 10.1107/S1600536810032265/pk2258sup1.cif

Structure factors: contains datablocks I. DOI: 10.1107/S1600536810032265/pk2258Isup2.hkl

Additional supplementary materials:  crystallographic information; 3D view; checkCIF report

## Figures and Tables

**Table 1:** Hydrogen-bond geometry (Å, °)

*D*—H⋯*A*	*D*—H	H⋯*A*	*D*⋯*A*	*D*—H⋯*A*
O3—H3O⋯O4	0.84	1.76	2.509 (3)	147

**Fig. 1.**
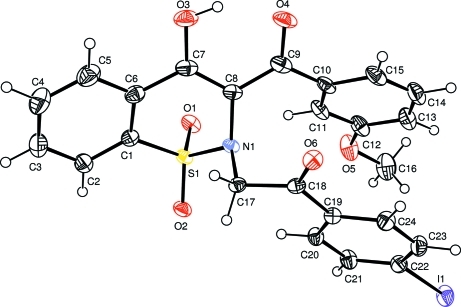
The title molecule plotted with the displacement ellipsoids at 50% probability level (Farrugia, 1997).

## supplementary crystallographic information

### Comment

Benzothiazine nucleus occupies a significant position among heterocyclic compounds. Oxicams are benzothiazine derivatives which are in use for the treatment of various inflammatory diseases (Lombardino & Wiseman, 1972; Zinnes *et al.*, 1982). Besides oxicams, numerous other benzothiazine compounds are found to possess antimicrobial (Zia-ur-Rehman *et al.*, 2005), analgesic (Turck *et al.*, 1996), antioxidant activities (Ahmad *et al.*, 2010). In this paper, we report the synthesis and crystal structure of the title compound.

The structure of the title compound contains independent molecules separated by normal van der Waals distances (Fig. 1). The heterocyclic thiazine ring adopts a half-chair conformation, with atoms S1 and N1 displaced by 0.381 (5) and -0.449 (5) Å, respectively, from the plane formed by atoms C1/C6/C7/C8; the puckering parameters (Cremer & Pople, 1975) are: *Q* = 0.550 (2) Å, θ = 61.7 (2)° and φ = 31.4 (3)°. Similar conformations of the corresponding rings have been reported in some closely related compounds (Siddiqui *et al.*, 2008). Unlike the structure of 4-hydroxy-3-(3-methoxy)benzoyl-2-(3-methoxy)phenacyl-2*H*-1,2- benzothiazine 1,1-dioxide (Gul *et al.*, 2010) where in the carbon fragments C1–C15 and C17–C24 were more or less planar individually and lie at an angle 77.17 (2)° with respect to each other, the benzene rings C10–C15 and C19–C24 in the title compound, lie almost parallel to each other (dihedral angle 9.18 (4)°) with a separation of 3.754 (2) Å between the centers of gravity of the two rings indicating strong π–π interactions.

The structure is stabilized by intramolecular interactions C11—H11···N1 and O3—H3O···O4 resulting in six membered rings and C17—H17A···O2 forming a five membered ring (Table 1).

### Experimental

4-Hydroxy-1,1-dioxido-2*H*-1,2-benzothiazin-3-yl)(3-methoxyphenyl) methanone (2.0 g, 6.0 mmol), K_2_CO_3_ (1.24 g, 9.0 mmol), 4-iodophenacyl bromide (2.01 g, 6.2 mmol) and acetonitrile (25 ml) were refluxed for 6 h. The completion of reaction was monitored by TLC. After cooling to room temperature, the reaction mixture was poured into ice cold water. Yellow precipitates of the title compound obtained were filtered, washed with cold water and dried. The crystals suitable for X-ray crystallographic analysis were grown from a solution of methanol and chloroform (1:1).

### Refinement

The H-atoms were located from difference Fourier maps and were included in the refinement at geometrically idealized positions in riding-model approximation with O—H = 0.84 Å and C—H = 0.95–0.99 Å; the *U*_iso_(H) were allowed at 1.2*U*_eq_(C) or 1.5*U*_eq_(O). The final difference map was essentially featurless.

### Crystal data

**Table:**

C_24_H_18_INO_6_S	*F*(000) = 1144
*M**_r_* = 575.35	*D*_x_ = 1.692 Mg m^−^^3^
Monoclinic, *P*2_1_/*c*	Mo *K*α radiation, λ = 0.71073 Å
Hall symbol: -P 2ybc	Cell parameters from 5266 reflections
*a* = 9.7392 (2) Å	θ = 1.0–27.5°
*b* = 11.5288 (3) Å	µ = 1.55 mm^−^^1^
*c* = 20.4634 (4) Å	*T* = 173 K
β = 100.5288 (11)°	Prism, yellow
*V* = 2258.97 (9) Å^3^	0.12 × 0.10 × 0.08 mm
*Z* = 4	

### Data collection

**Table:**

Nonius KappaCCD diffractometer	3960 independent reflections
Radiation source: fine-focus sealed tube	3620 reflections with *I* > 2σ(*I*)
graphite	*R*_int_ = 0.017
ω and φ scans	θ_max_ = 25.0°, θ_min_ = 2.7°
Absorption correction: multi-scan (*SORTAV*; Blessing, 1997)	*h* = −11→11
*T*_min_ = 0.836, *T*_max_ = 0.886	*k* = −13→13
16229 measured reflections	*l* = −24→24

### Refinement

**Table:**

Refinement on *F*^2^	Primary atom site location: structure-invariant direct methods
Least-squares matrix: full	Secondary atom site location: difference Fourier map
*R*[*F*^2^ > 2σ(*F*^2^)] = 0.026	Hydrogen site location: difference Fourier map
*wR*(*F*^2^) = 0.061	H-atom parameters constrained
*S* = 1.08	*w* = 1/[σ^2^(*F*_o_^2^) + (0.0174*P*)^2^ + 2.9397*P*] where *P* = (*F*_o_^2^ + 2*F*_c_^2^)/3
3960 reflections	(Δ/σ)_max_ = 0.001
300 parameters	Δρ_max_ = 0.76 e Å^−^^3^
0 restraints	Δρ_min_ = −0.83 e Å^−^^3^

### Special details

**Table:**

Experimental. Yield: 1.96 g, 70%, m.p. 413–414 K, IR (KBr, ν_max_): 2957, 1682, 1340, 1128 cm^-1^, EI–MS (*m/z*): 575.0
Geometry. All e.s.d.'s (except the e.s.d. in the dihedral angle between two l.s. planes) are estimated using the full covariance matrix. The cell e.s.d.'s are taken into account individually in the estimation of e.s.d.'s in distances, angles and torsion angles; correlations between e.s.d.'s in cell parameters are only used when they are defined by crystal symmetry. An approximate (isotropic) treatment of cell e.s.d.'s is used for estimating e.s.d.'s involving l.s. planes.
Refinement. Refinement of *F*^2^ against ALL reflections. The weighted *R*-factor *wR* and goodness of fit *S* are based on *F*^2^, conventional *R*-factors *R* are based on *F*, with *F* set to zero for negative *F*^2^. The threshold expression of *F*^2^ > σ(*F*^2^) is used only for calculating *R*-factors(gt) *etc*. and is not relevant to the choice of reflections for refinement. *R*-factors based on *F*^2^ are statistically about twice as large as those based on *F*, and *R*- factors based on ALL data will be even larger.

### Fractional atomic coordinates and isotropic or equivalent isotropic displacement parameters (Å^2^)

**Table:**

	*x*	*y*	*z*	*U*_iso_*/*U*_eq_	
I1	0.309451 (19)	0.222186 (19)	0.434249 (8)	0.04263 (8)	
S1	−0.12938 (6)	0.42205 (5)	0.72012 (3)	0.02558 (14)	
O1	−0.15136 (17)	0.53974 (15)	0.69887 (9)	0.0335 (4)	
O2	−0.18899 (17)	0.33084 (16)	0.67691 (9)	0.0330 (4)	
O3	0.1160 (2)	0.56345 (19)	0.89527 (10)	0.0465 (5)	
H3O	0.1921	0.5875	0.8867	0.070*	
O4	0.30729 (19)	0.59168 (17)	0.82968 (11)	0.0448 (5)	
O5	0.1389 (2)	0.5711 (2)	0.54065 (11)	0.0515 (6)	
O6	0.33468 (18)	0.27269 (16)	0.77976 (9)	0.0310 (4)	
N1	0.04075 (19)	0.40214 (17)	0.73891 (10)	0.0239 (4)	
C1	−0.1770 (3)	0.4068 (2)	0.79852 (12)	0.0281 (5)	
C2	−0.3034 (3)	0.3567 (2)	0.80468 (14)	0.0346 (6)	
H2	−0.3641	0.3266	0.7668	0.042*	
C3	−0.3395 (3)	0.3513 (3)	0.86691 (16)	0.0465 (7)	
H3	−0.4260	0.3178	0.8720	0.056*	
C4	−0.2503 (4)	0.3946 (3)	0.92140 (17)	0.0563 (9)	
H4	−0.2765	0.3917	0.9639	0.068*	
C5	−0.1229 (3)	0.4422 (3)	0.91516 (15)	0.0495 (8)	
H5	−0.0621	0.4705	0.9535	0.059*	
C6	−0.0831 (3)	0.4489 (2)	0.85358 (13)	0.0334 (6)	
C7	0.0526 (3)	0.4997 (2)	0.84576 (14)	0.0343 (6)	
C8	0.1083 (2)	0.4833 (2)	0.78869 (13)	0.0277 (5)	
C9	0.2346 (3)	0.5410 (2)	0.78018 (15)	0.0350 (6)	
C10	0.2832 (3)	0.5437 (2)	0.71554 (15)	0.0337 (6)	
C11	0.1901 (3)	0.5553 (2)	0.65576 (15)	0.0359 (6)	
H11	0.0927	0.5602	0.6556	0.043*	
C12	0.2394 (3)	0.5599 (2)	0.59636 (16)	0.0403 (7)	
C13	0.3816 (3)	0.5525 (3)	0.59645 (17)	0.0448 (7)	
H13	0.4154	0.5543	0.5557	0.054*	
C14	0.4742 (3)	0.5427 (3)	0.65634 (17)	0.0461 (8)	
H14	0.5716	0.5386	0.6564	0.055*	
C15	0.4271 (3)	0.5388 (2)	0.71568 (16)	0.0403 (7)	
H15	0.4915	0.5328	0.7564	0.048*	
C16	0.1815 (4)	0.5671 (4)	0.47757 (17)	0.0605 (10)	
H16A	0.0988	0.5674	0.4422	0.073*	
H16B	0.2355	0.4963	0.4745	0.073*	
H16C	0.2394	0.6350	0.4727	0.073*	
C17	0.0871 (3)	0.2799 (2)	0.75191 (12)	0.0251 (5)	
H17A	0.0214	0.2265	0.7240	0.030*	
H17B	0.0890	0.2602	0.7992	0.030*	
C18	0.2332 (3)	0.2673 (2)	0.73533 (12)	0.0250 (5)	
C19	0.2470 (3)	0.2523 (2)	0.66453 (12)	0.0256 (5)	
C20	0.1333 (3)	0.2615 (2)	0.61251 (13)	0.0292 (6)	
H20	0.0426	0.2741	0.6220	0.035*	
C21	0.1508 (3)	0.2524 (2)	0.54714 (13)	0.0334 (6)	
H21	0.0731	0.2603	0.5119	0.040*	
C22	0.2828 (3)	0.2318 (2)	0.53356 (13)	0.0313 (6)	
C23	0.3973 (3)	0.2196 (3)	0.58470 (14)	0.0355 (6)	
H23	0.4872	0.2037	0.5750	0.043*	
C24	0.3789 (3)	0.2308 (2)	0.64959 (13)	0.0320 (6)	
H24	0.4572	0.2238	0.6847	0.038*	

### Atomic displacement parameters (Å^2^)

**Table:**

	*U*^11^	*U*^22^	*U*^33^	*U*^12^	*U*^13^	*U*^23^
I1	0.03981 (12)	0.06393 (15)	0.02580 (11)	−0.00825 (9)	0.01039 (8)	−0.00513 (8)
S1	0.0217 (3)	0.0240 (3)	0.0290 (3)	−0.0021 (2)	−0.0009 (2)	0.0009 (2)
O1	0.0263 (9)	0.0271 (9)	0.0435 (11)	0.0010 (7)	−0.0030 (8)	0.0076 (8)
O2	0.0305 (9)	0.0333 (10)	0.0335 (10)	−0.0090 (8)	0.0016 (8)	−0.0047 (8)
O3	0.0362 (11)	0.0499 (13)	0.0488 (12)	−0.0011 (10)	−0.0046 (9)	−0.0239 (10)
O4	0.0290 (10)	0.0373 (11)	0.0609 (13)	−0.0048 (8)	−0.0103 (9)	−0.0153 (10)
O5	0.0337 (10)	0.0716 (16)	0.0476 (13)	0.0016 (10)	0.0034 (9)	0.0235 (11)
O6	0.0295 (9)	0.0363 (10)	0.0248 (9)	0.0047 (8)	−0.0017 (8)	0.0009 (8)
N1	0.0211 (10)	0.0205 (10)	0.0281 (11)	−0.0006 (8)	−0.0011 (8)	−0.0003 (8)
C1	0.0288 (13)	0.0257 (13)	0.0294 (13)	0.0046 (10)	0.0040 (10)	0.0006 (10)
C2	0.0311 (13)	0.0314 (15)	0.0417 (15)	0.0026 (11)	0.0074 (12)	0.0027 (12)
C3	0.0421 (16)	0.0507 (19)	0.0509 (18)	0.0010 (15)	0.0194 (14)	0.0049 (15)
C4	0.062 (2)	0.072 (2)	0.0396 (17)	0.0041 (18)	0.0203 (16)	0.0002 (17)
C5	0.0495 (18)	0.061 (2)	0.0362 (16)	0.0040 (16)	0.0044 (14)	−0.0104 (15)
C6	0.0328 (13)	0.0322 (15)	0.0341 (14)	0.0061 (11)	0.0029 (11)	−0.0045 (11)
C7	0.0291 (13)	0.0288 (14)	0.0403 (15)	0.0056 (11)	−0.0066 (11)	−0.0076 (12)
C8	0.0231 (12)	0.0223 (12)	0.0340 (14)	0.0038 (10)	−0.0048 (10)	−0.0031 (11)
C9	0.0256 (13)	0.0203 (13)	0.0540 (17)	0.0053 (10)	−0.0059 (12)	−0.0003 (12)
C10	0.0239 (12)	0.0187 (13)	0.0559 (17)	−0.0031 (10)	0.0004 (12)	0.0030 (12)
C11	0.0221 (12)	0.0288 (14)	0.0542 (17)	−0.0044 (11)	0.0001 (12)	0.0113 (13)
C12	0.0321 (14)	0.0317 (15)	0.0551 (18)	−0.0028 (12)	0.0030 (13)	0.0171 (13)
C13	0.0318 (14)	0.0421 (17)	0.062 (2)	−0.0022 (13)	0.0123 (14)	0.0108 (15)
C14	0.0235 (13)	0.0397 (17)	0.074 (2)	−0.0025 (12)	0.0065 (14)	0.0071 (16)
C15	0.0230 (13)	0.0295 (15)	0.063 (2)	−0.0016 (11)	−0.0053 (13)	0.0036 (14)
C16	0.0512 (19)	0.078 (3)	0.053 (2)	0.0050 (18)	0.0125 (16)	0.0238 (19)
C17	0.0303 (13)	0.0198 (12)	0.0252 (12)	0.0014 (10)	0.0049 (10)	−0.0005 (10)
C18	0.0303 (13)	0.0178 (12)	0.0262 (13)	0.0029 (10)	0.0033 (11)	0.0017 (10)
C19	0.0270 (12)	0.0248 (13)	0.0239 (12)	0.0013 (10)	0.0019 (10)	0.0019 (10)
C20	0.0249 (12)	0.0365 (15)	0.0259 (13)	−0.0008 (11)	0.0035 (10)	0.0013 (11)
C21	0.0265 (13)	0.0463 (16)	0.0254 (13)	−0.0035 (12)	−0.0007 (10)	0.0014 (12)
C22	0.0337 (14)	0.0378 (15)	0.0230 (13)	−0.0045 (12)	0.0072 (11)	−0.0024 (11)
C23	0.0260 (13)	0.0472 (17)	0.0335 (15)	0.0013 (12)	0.0063 (11)	−0.0031 (13)
C24	0.0257 (13)	0.0403 (16)	0.0277 (14)	0.0034 (11)	−0.0014 (10)	−0.0014 (11)

### Geometric parameters (Å, °)

**Table:**

I1—C22	2.098 (3)	C10—C11	1.389 (4)
S1—O2	1.4272 (18)	C10—C15	1.402 (4)
S1—O1	1.4290 (18)	C11—C12	1.387 (4)
S1—N1	1.6472 (19)	C11—H11	0.9500
S1—C1	1.758 (3)	C12—C13	1.388 (4)
O3—C7	1.311 (3)	C13—C14	1.387 (4)
O3—H3O	0.8400	C13—H13	0.9500
O4—C9	1.267 (3)	C14—C15	1.375 (4)
O5—C12	1.366 (4)	C14—H14	0.9500
O5—C16	1.427 (4)	C15—H15	0.9500
O6—C18	1.216 (3)	C16—H16A	0.9800
N1—C8	1.450 (3)	C16—H16B	0.9800
N1—C17	1.489 (3)	C16—H16C	0.9800
C1—C2	1.386 (4)	C17—C18	1.530 (3)
C1—C6	1.402 (4)	C17—H17A	0.9900
C2—C3	1.383 (4)	C17—H17B	0.9900
C2—H2	0.9500	C18—C19	1.489 (3)
C3—C4	1.376 (5)	C19—C20	1.393 (3)
C3—H3	0.9500	C19—C24	1.396 (4)
C4—C5	1.383 (5)	C20—C21	1.383 (4)
C4—H4	0.9500	C20—H20	0.9500
C5—C6	1.387 (4)	C21—C22	1.384 (4)
C5—H5	0.9500	C21—H21	0.9500
C6—C7	1.480 (4)	C22—C23	1.390 (4)
C7—C8	1.388 (4)	C23—C24	1.378 (4)
C8—C9	1.437 (4)	C23—H23	0.9500
C9—C10	1.484 (4)	C24—H24	0.9500
			
O2—S1—O1	119.32 (11)	O5—C12—C13	124.7 (3)
O2—S1—N1	108.66 (10)	C11—C12—C13	120.1 (3)
O1—S1—N1	106.96 (10)	C14—C13—C12	119.6 (3)
O2—S1—C1	110.27 (12)	C14—C13—H13	120.2
O1—S1—C1	108.82 (12)	C12—C13—H13	120.2
N1—S1—C1	101.24 (11)	C15—C14—C13	121.0 (3)
C7—O3—H3O	109.5	C15—C14—H14	119.5
C12—O5—C16	118.0 (2)	C13—C14—H14	119.5
C8—N1—C17	113.69 (18)	C14—C15—C10	119.5 (3)
C8—N1—S1	112.47 (15)	C14—C15—H15	120.3
C17—N1—S1	115.63 (15)	C10—C15—H15	120.3
C2—C1—C6	122.1 (2)	O5—C16—H16A	109.5
C2—C1—S1	120.8 (2)	O5—C16—H16B	109.5
C6—C1—S1	117.17 (19)	H16A—C16—H16B	109.5
C3—C2—C1	118.8 (3)	O5—C16—H16C	109.5
C3—C2—H2	120.6	H16A—C16—H16C	109.5
C1—C2—H2	120.6	H16B—C16—H16C	109.5
C4—C3—C2	120.0 (3)	N1—C17—C18	108.30 (19)
C4—C3—H3	120.0	N1—C17—H17A	110.0
C2—C3—H3	120.0	C18—C17—H17A	110.0
C3—C4—C5	120.9 (3)	N1—C17—H17B	110.0
C3—C4—H4	119.6	C18—C17—H17B	110.0
C5—C4—H4	119.6	H17A—C17—H17B	108.4
C4—C5—C6	120.7 (3)	O6—C18—C19	121.9 (2)
C4—C5—H5	119.6	O6—C18—C17	119.4 (2)
C6—C5—H5	119.6	C19—C18—C17	118.7 (2)
C5—C6—C1	117.5 (3)	C20—C19—C24	118.7 (2)
C5—C6—C7	121.6 (3)	C20—C19—C18	122.3 (2)
C1—C6—C7	120.9 (2)	C24—C19—C18	118.9 (2)
O3—C7—C8	121.7 (2)	C21—C20—C19	120.8 (2)
O3—C7—C6	116.2 (2)	C21—C20—H20	119.6
C8—C7—C6	122.1 (2)	C19—C20—H20	119.6
C7—C8—C9	120.9 (2)	C20—C21—C22	119.4 (2)
C7—C8—N1	118.8 (2)	C20—C21—H21	120.3
C9—C8—N1	120.2 (2)	C22—C21—H21	120.3
O4—C9—C8	118.8 (3)	C21—C22—C23	120.9 (2)
O4—C9—C10	118.7 (2)	C21—C22—I1	119.18 (19)
C8—C9—C10	122.5 (2)	C23—C22—I1	119.96 (19)
C11—C10—C15	119.8 (3)	C24—C23—C22	119.2 (2)
C11—C10—C9	121.6 (2)	C24—C23—H23	120.4
C15—C10—C9	118.6 (3)	C22—C23—H23	120.4
C12—C11—C10	120.1 (2)	C23—C24—C19	121.0 (2)
C12—C11—H11	120.0	C23—C24—H24	119.5
C10—C11—H11	120.0	C19—C24—H24	119.5
O5—C12—C11	115.2 (2)		
			
O2—S1—N1—C8	−173.39 (16)	C7—C8—C9—C10	−168.2 (2)
O1—S1—N1—C8	56.56 (19)	N1—C8—C9—C10	14.9 (4)
C1—S1—N1—C8	−57.30 (19)	O4—C9—C10—C11	−141.7 (3)
O2—S1—N1—C17	−40.51 (19)	C8—C9—C10—C11	38.2 (4)
O1—S1—N1—C17	−170.56 (17)	O4—C9—C10—C15	35.8 (4)
C1—S1—N1—C17	75.58 (18)	C8—C9—C10—C15	−144.3 (3)
O2—S1—C1—C2	−30.4 (2)	C15—C10—C11—C12	1.2 (4)
O1—S1—C1—C2	102.2 (2)	C9—C10—C11—C12	178.7 (3)
N1—S1—C1—C2	−145.3 (2)	C16—O5—C12—C11	−175.2 (3)
O2—S1—C1—C6	150.53 (19)	C16—O5—C12—C13	4.5 (4)
O1—S1—C1—C6	−76.8 (2)	C10—C11—C12—O5	179.8 (2)
N1—S1—C1—C6	35.6 (2)	C10—C11—C12—C13	0.1 (4)
C6—C1—C2—C3	2.0 (4)	O5—C12—C13—C14	179.2 (3)
S1—C1—C2—C3	−177.0 (2)	C11—C12—C13—C14	−1.1 (4)
C1—C2—C3—C4	−0.4 (5)	C12—C13—C14—C15	0.7 (5)
C2—C3—C4—C5	−1.0 (5)	C13—C14—C15—C10	0.6 (4)
C3—C4—C5—C6	0.9 (5)	C11—C10—C15—C14	−1.6 (4)
C4—C5—C6—C1	0.6 (5)	C9—C10—C15—C14	−179.2 (3)
C4—C5—C6—C7	179.9 (3)	C8—N1—C17—C18	−74.6 (2)
C2—C1—C6—C5	−2.0 (4)	S1—N1—C17—C18	153.04 (16)
S1—C1—C6—C5	177.0 (2)	N1—C17—C18—O6	97.6 (3)
C2—C1—C6—C7	178.7 (2)	N1—C17—C18—C19	−80.6 (3)
S1—C1—C6—C7	−2.3 (3)	O6—C18—C19—C20	−171.2 (2)
C5—C6—C7—O3	−16.8 (4)	C17—C18—C19—C20	6.9 (3)
C1—C6—C7—O3	162.5 (2)	O6—C18—C19—C24	7.4 (4)
C5—C6—C7—C8	164.7 (3)	C17—C18—C19—C24	−174.5 (2)
C1—C6—C7—C8	−16.0 (4)	C24—C19—C20—C21	−1.6 (4)
O3—C7—C8—C9	−3.4 (4)	C18—C19—C20—C21	177.0 (2)
C6—C7—C8—C9	175.0 (2)	C19—C20—C21—C22	1.3 (4)
O3—C7—C8—N1	173.5 (2)	C20—C21—C22—C23	0.3 (4)
C6—C7—C8—N1	−8.0 (4)	C20—C21—C22—I1	−178.5 (2)
C17—N1—C8—C7	−85.5 (3)	C21—C22—C23—C24	−1.4 (4)
S1—N1—C8—C7	48.3 (3)	I1—C22—C23—C24	177.4 (2)
C17—N1—C8—C9	91.4 (3)	C22—C23—C24—C19	1.0 (4)
S1—N1—C8—C9	−134.7 (2)	C20—C19—C24—C23	0.5 (4)
C7—C8—C9—O4	11.6 (4)	C18—C19—C24—C23	−178.2 (2)
N1—C8—C9—O4	−165.2 (2)		

### Hydrogen-bond geometry (Å, °)

**Table:**

*D*—H···*A*	*D*—H	H···*A*	*D*···*A*	*D*—H···*A*
O3—H3O···O4	0.84	1.76	2.509 (3)	147
C11—H11···N1	0.95	2.61	3.006 (3)	106
C17—H17A···O2	0.99	2.42	2.902 (3)	109

## References

[bb1] Ahmad, M., Siddiqui, H. L., Zia-ur-Rehman, M. & Parvez, M. (2010). *Eur. J. Med. Chem.* **45**, 698–704. 10.1016/j.ejmech.2009.11.016 19962218

[bb2] Blessing, R. H. (1997). *J. Appl. Cryst.* **30**, 421–426.

[bb3] Cremer, D. & Pople, J. A. (1975). *J. Am. Chem. Soc.* **97**, 1354–1358.

[bb4] Farrugia, L. J. (1997). *J. Appl. Cryst.* **30**, 565.

[bb5] Gul, S., Siddiqui, H. L., Ahmad, M., Nisar, M. & Parvez, M. (2010). *Acta Cryst.* E**66**, o2314–o2315. 10.1107/S1600536810031673 PMC3007862 21588662

[bb6] Lombardino, J. G. & Wiseman, E. H. (1972). *J. Med. Chem.* **15**, 848–849. 10.1021/jm00278a016 4625532

[bb7] Nonius (1998). *COLLECT* Nonius BV, Delft, The Netherlands.

[bb8] Otwinowski, Z. & Minor, W. (1997). *Methods in Enzymology*, Vol. 276, *Macromolecular Crystallography*, Part A, edited by C. W. Carter Jr & R. M. Sweet, pp. 307–326. New York: Academic Press.

[bb9] Sheldrick, G. M. (2008). *Acta Cryst.* A**64**, 112–122. 10.1107/S0108767307043930 18156677

[bb10] Siddiqui, W. A., Ahmad, S., Tariq, M. I., Siddiqui, H. L. & Parvez, M. (2008). *Acta Cryst.* C**64**, o4–o6. 10.1107/S0108270107059173 18216455

[bb11] Turck, D., Busch, U., Heinzel, G., Narjes, H. & Nehmiz, G. (1996). *J. Clin. Pharmacol.* **36**, 79–84. 10.1002/j.1552-4604.1996.tb04155.x 8932547

[bb12] Zia-ur-Rehman, M., Choudary, J. A. & Ahmad, S. (2005). *Bull. Korean Chem. Soc.* **54**, 1171–1175. 10.1248/cpb.54.1175 16880664

[bb13] Zinnes, H., Sircar, J. C., Lindo, N., Schwartz, M. L., Fabian, A. C., Shavel, J. Jr, Kasulanis, C. F., Genzer, J. D., Lutomski, C. & DiPasquale, G. (1982). *J. Med. Chem.* **25**, 12–18. 10.1021/jm00343a003 7086815

